# Association of Eating Disorders in Prenatal and Perinatal Women and Its Complications in Their Offspring

**DOI:** 10.7759/cureus.31429

**Published:** 2022-11-12

**Authors:** Sushmita Dutta, Prasad Deshmukh

**Affiliations:** 1 Obstetrics and Gynaecology, Jawaharlal Nehru Medical College, Datta Meghe Institute of Medical Sciences, Wardha, IND; 2 Otolaryngology-Head and Neck Surgery, Jawaharlal Nehru Medical College, Datta Meghe Institute of Medical Sciences, Wardha, IND

**Keywords:** anorexia nervosa, postpartum distress, overweight, emotional disturbances, neurodevelopmental disorders

## Abstract

Numerous studies revealed that women in the first trimester of pregnancy (prenatal) and 6-12 months after delivery of a newborn (postnatal) suffer from eating disorders like anorexia nervosa, bulimia nervosa, and binge eating disorder. Pregnancy may increase or decrease the symptoms of eating disorders. It varies from person to person. The mother faces many complications during this period which may also affect the newborn child. Weight loss is an essential symptom of eating disorders, which may cause extreme anxiety and depression during and after pregnancy. Stress is another symptom that is associated with binge eating disorders. The main aim of this narrative review article is to critically analyze and discuss the association of prenatal and postnatal factors that lead to different eating disorders in the mother and child. A total of 38 published and standard articles were selected for this review. The studies under consideration showed numerous methodological shortcomings, necessitating additional investigation to explain these discrepancies. The evidence points to a connection between prenatal and perinatal variables, and the children of these women also develop eating disorders. Earlier research focused on linking pregnancy and eating disorders, mainly emphasizing anorexia nervosa and bulimia nervosa. However, any significant correlation between binge eating disorder and pregnancy is yet to be found. In the first half of pregnancy, about 33%-35% of women who had binge eating disorder before becoming pregnant no longer met the diagnostic criteria, their illness improved, and they seemed to recover. These patients with eating disorders are more likely to have pregnancy-related complications and births. Therefore, creating a multidisciplinary screening strategy and guidelines for managing and supervising this particular patient population makes sense.

## Introduction and background

Eating disorders can be considered one of the major concerns of pregnancy, affecting the overall health of both mother and child [1]. A collection of mental health conditions known as eating disorders are characterized by eating behavior disruptions that can sometimes gravely harm one's physical and psychological well-being [2]. The most common eating disorders in pregnancy are anorexia nervosa (AN), bulimia nervosa (BN), and binge-eating disorder (BED) [3]. The eating disorder most commonly affects women of the reproductive age group and also may affect 5.1%-7.5% of women during pregnancy. During pregnancy, eating disorder symptoms typically lessen in females already suffering from eating disorders. However, there is evidence of postnatal relapse and persistent symptoms [4]. Additionally, postpartum distress and anxiety symptoms are frequent for women with present or former eating disorders [5].

Although eating disorders are common among women of childbearing age, there has, to our knowledge, been little research done on how they may affect pregnancy and the problems related to the health of both mother and newborn [6]. Numerous research has connected perinatal and prenatal issues with eating disorders, with the idea that minor neurological impairments, neuropsychological limitations, and irreversible morphological brain abnormalities might accelerate the start of eating disorders [7].

Mixed results were found in a descriptive analysis of 22 publications published more recently that focused on other risk factors (including the function of sex hormones, maternal status, and maternal eating disorders). An alternative analysis of 13 research studies used the concept of "fetal programming" to explain how stimuli or insults received during sensitive or important stages of embryonic development may have metabolic effects that last a lifetime. The subsequent review again disputed findings and included new risk variables, namely maternal stress during pregnancy. Therefore, this systematic review had two objectives (a) enhance knowledge of the crucial concerns that future types of research must address, and (b) critically analyze the most recent research on eating disorders in pregnancy, their complications during childbirth, and the start of eating disorders in their offspring [7]. The media is also interested in this topic; in 2008, Fox News and The Early Show journalists discussed the emergence of a phenomenon involving restrictive behaviors among pregnant women, such as calorie restriction and increased exercise to maintain a perfect figure both during pregnancy and right after delivery [8].

## Review

The Mixed Methods Appraisal Tool (MMAT) reviewed the publications included in this systematic review, and the PRISMA statement standards were used to create this review [9]. The different articles have been searched and read thoroughly from reliable sources like PubMed and Google Scholar. Many topics are aggregated together, and an accurate outcome/result has been made. All the full-text review articles were included if they met the concerned criteria.

Factors affecting eating disorders

Throughout the prenatal and postpartum phases of pregnancy, various factors give rise to several eating disorders in their offspring. The major maternal factors associated with eating disorders in their offspring are - maternal age - a registered study discovered that adolescents whose mothers were young when they delivered a baby had a lesser chance of getting eating disorders compared to adolescents whose mothers were between the ages of 25 and 28 when they gave birth.

Maternal weight: There is no significant association between the mother's body mass index and the start of AN or BN in their offspring. On the contrary, a current study discovered that maternal obesity and overweight reduced the incidence of eating disorders in female-born children at term in a dose-response fashion [10].

Cigarette smoking and alcohol consumption: Even after excluding confounding variables like adult BMI of the offspring or differences in BMI of a child and adult, parental smoking and the diagnosis of BN in their offspring are related. A considerable negative connection between AN and a mother's smoking was discovered in a different study; however, this was significantly diminished when parental education was taken into account [10].

Maternal stress during pregnancy: In a large population-based cohort research, it was discovered that girls whose mothers lost a close relative from the year before conception through the end of their pregnancy had a higher risk of developing an ED than healthy controls. The findings were similar for mixed eating disorders and BN but not for AN [10].

Pregnancy-related concerns

Detection of postnatal depression: The community was screened for postnatal depression using a 10-item self-report questionnaire called the Edinburgh Postnatal Depression Scale (EPDS). Using the research diagnostic criteria for depressive illness obtained from Goldberg's Standardized Psychiatric Interview, 84 mothers were the subjects of validation research following extensive pilot interviews. It was discovered that the EPDS had good sensitivity and specificity and was also responsive to changes in the severity of depression over time. The scale features an easy scoring process and can be finished in around five minutes. In the secondary prevention of postnatal depression, the EPDS score is employed. Eating disorders were evaluated using the eating disorder examination questionnaire, while anxiety and depression symptoms were evaluated using the primary care evaluation of mental disorders. Additionally evaluated were pre-gestational weight and body mass index [11].

Emotional disturbances: Prenatal care providers for females suffering from eating disorders need to be aware that the perinatal period is particularly vulnerable because it is a time of profound physical and psychosocial change [11]. When they found out they were pregnant, negative feelings were more likely seen in women with eating disorders. These emotions seem to last for at least 18 weeks in females suffering from AN and BN, if not longer [12]. Additionally, there is failure anxiety, a change in food habits and body image, and concerns about the child's physical development and emotional control. To appropriately treat issues specific to women who experience an eating problem during the perinatal period, compassionate and thoughtful care is required [13].

Eating behaviors and patterns: According to much other research, pregnancy can cause eating problem relapse in women who are in recovery [14]. According to one study, 33% of pregnant females suffering from AN history experienced a recurrence of symptoms, necessitating consultation with a mental health professional (though none required hospitalization) [15]. Another study found that pregnant women who had previously struggled with eating disorders had concerns about their weight and shape, and eating disorder behaviors had increased in such women by certain behaviors like restricting food intake and performing intense exercise [16].

Weight gain: While substantial weight gain during pregnancy is regarded to be protective in females suffering from AN, patients with BN and BED were even more likely to experience it [17]. Increased prenatal weight gain in the Norwegian Mother and Child Cohort Study (MoBa) may be due to continuous binge eating in women with BN, eating disorder not otherwise specified (EDNOS-purging type), and BED. However, it may also be due to greater purging behavior control in these women [18].

Associated factors of anxiety and depression: During COVID-19, gestational age, eating behavior, smoking, and physical activity were put into consideration, and it was seen that the pattern of eating habits got affected during COVID-19. This was also related to depression in such patients [19].

Impact on breastfeeding practices

According to recent scientific research, breastfeeding is a reliable method for preserving and enhancing baby health. In any event, it can be said that mothers with any eating disorder subtype are likely to experience more breastfeeding-related issues, which is difficult for both the mother and the child [20]. The child may also experience these negative emotions and communicate their discomfort by acting differently. According to recent research, oxytocin may play a role in both the emergence of eating disorders and the control of eating behavior. The neurohormone oxytocin, which affects eating behavior as well as cognitive, emotional, and social functioning, is crucial to the pathophysiology of eating disorders, which can affect people of all weight spectra. According to recent research, the oxytocin system plays a role in the pathophysiology of AN. Endogenous oxytocin levels in BN patients were comparable to those in healthy controls, while exogenous oxytocin decreased food consumption. Breastfeeding issues experienced by mothers with disordered eating habits may be partially explained by oxytocin functioning, and it may even play a role in the intergenerational transmission of eating disorders via early-life attachment experiences. Studies in this context should take the manner of delivery into account because cesarean sections modify the levels of the hormone oxytocin after birth, which may affect the mother's breastfeeding practices [21]. Mothers face tremendous social pressure and the desire to breastfeed their children regardless of why they are unable to or choose not to do so. Other mental illnesses, such as prenatal mood and anxiety disorders, also coexist with eating disorders. Future research evaluating breastfeeding women with eating disorders should take these factors into careful consideration as they may affect the decision to start, continue, or discontinue breastfeeding [21].

Correlation of eating disorders in childbearing women with epilepsy and their dietary pattern

When compared to pregnant women without epilepsy, women with epilepsy had a considerably higher risk of BED and "impaired body image." The use of anti-epileptic drugs is thought to be a mediator of unfavorable birth outcomes in women with epilepsy (WWE). At the same time, the precise mechanisms of action and the function of confounding variables are still unknown. Comorbid eating disorders are a potential cause of pregnancy problems in women with epilepsy that has not yet been thoroughly investigated [22]. Pre-eclampsia, gestational hyperglycemia, and pregnancy-induced hypertension were all more common in BED patients. Extended labor was more common, and pregnant women with eating disorders were more likely to have a cesarean section. Their newborns had a lower appearance, pulse, grimace, activity, and respiration (APGAR) scores. They more frequently experienced perinatal mortality and were more likely to need CPR [22]. Therefore, in consultations both before and during pregnancy, it is important to take into account and reduce the higher risk of complications in WWE with ED and potential negative health effects on the mother and child. Currently, eating disorders are a health issue that has a significant negative influence on society. Studies conducted in the community are crucial since few people with eating disorders seek treatment [23]. Mothers with eating disorders were shown to eat less meat and more soy and bean products. On the "vegetarian" dietary pattern, they received higher scores (high intakes of meat alternatives, pulses, nuts, and herbal teas and high negative intakes of red meat and poultry). Additionally, they had lower consumption rates of butter, whole milk, sweets, and saturated fats. Their macronutrient, vitamin, and mineral intake were appropriate [24]. It has been determined that there were no variations in breastfeeding onset and cessation between females suffering from eating disorders and healthy mothers [25]. The related complications are depicted in Table 1 [26].

**Table 1:** Complexities in mother and child due to eating disorders. BED: Binge eating disorder, AN: Anorexia nervosa, BN: Bulimia nervosa, APGAR: Appearance, pulse, grimace, activity, and respiration Above mentioned are the complications of eating disorders in pregnant women and their newborns [26].

Eating Disorders	Complication in mother	Complication in newborn
BED	Preeclampsia, gestational hypertension, pregnancy-induced diabetes, increased rate of stillbirth, longer duration of 1st and 2nd stages of labor PCOS	Birth length longer than usual
AN	Postpartum hemorrhage	Birth length shorter than usual, lower birth weight
BN	Preeclampsia, gestational diabetes, more frequent abortions, polycystic ovary syndrome, induced labor	Low APGAR score at 1 min after birth

Clinical changes 

Amenorrhea: According to the most recent diagnostic guidelines, amenorrhea is a significant component of the features that are presented clinically. About 68%-89% of adult women patients with AN confirm that the condition has been present for at least three months of the disease. These forms of endocrine disruptions, which cause monthly cycle irregularities, are brought on by insufficient calorie intake and/or excessive exercise. The theory in the literature is that anorexia at least somewhat hampers conception because of changes in the anatomy and physiology of the female reproductive system during AN [26].

Maternal and fetal well-being: Pregnancy can be extremely challenging for women with eating disorders, especially in the initial stages of pregnancy. Patients' bodies go through changes during this early stage, but they are not yet noticeable enough to identify pregnancy. This proves to be a particularly challenging time for women with an eating disorder. As a result, it is crucial to consider how pregnancy affects ED progression as well as how the eating disorder affects the health of the mother and fetus [26].

Biomarkers of stress and nutrition in a pregnant woman with a history of eating disorders

Early-pregnant women with a history of AN had low levels of maternal serum ferritin, which is consistent with this group's high prevalence of anemia. Furthermore, in the combined group of patients and controls, there was a marginally favorable correlation between ferritin levels and the offspring's head circumference [27]. Other studies have discovered links between ferritin levels, both low and high, and several pregnancy problems, including preterm birth, preeclampsia, and intrauterine growth restriction [28]. Children with a history of eating disorders, particularly those with a history of AN, showed significantly lower motor, memory, language, and social skills at age five than children in the control group [29]. The results may not be indicative of the duration of pregnancy because the determination of serum biomarkers was limited to a single blood sample drawn during early pregnancy. The blood sample was not standardized and was not taken while the subject was fasting. Additionally, the sample size was small.

We discovered a strong relationship between maternal IGF-I and the child's head circumference in the sick group. Previous studies have shown a positive correlation between maternal serum IGF-I and childbirth weight. In agreement, our findings may help to confirm the contribution of IGF to fetal growth [30]. Various biomarkers of nutrition and stress related to pregnancy are given in Table 2 [30].

**Table 2:** Serum biomarkers of nutrition and stress with their normal values during first trimester of pregnancy TSH: Thyroid stimulating hormone, IGF-1: insulin-like growth factor 1, IGFBP1: Insulin like growth factor binding protein 1 Above mentioned are the serum biomarkers of nutrition and stress in pregnancy [30].

Serum Biomarkers	Normal range
1.Cortisol	7-19 mcg/dL
2.TSH	0.2-2.5 mU/L
3.INSULIN	95 mg/dL or less (before a meal) 120 mg/dL or less (2 hours after a meal)
4.Free T4	0.8-1.53 ng/dL
5.IGF-I	145 ng/mL
6.IGFBP1	26.3-76.8 ng/mL

Use of medication

Physicians may find it useful to know that consumption of psychotropic drugs, particularly anti-depressants, was high among females suffering from eating disorders during the pre-conception period as well as during pregnancy and postpartum [31]. For females suffering from eating disorders who are pregnant or contemplating pregnancy, evidence-based counseling regarding the benefit-risk balance of gestational exposure to anti-depressants or other psychotropic medicines and untreated psychiatric disease may be particularly crucial. The distinction between the impacts of eating disorders that have been treated and those that have not been treated is still mostly unknown. Most of the effects of eating disorders are still unclear, as opposed to those of eating disorders that have received treatment [32,33].

Because they have a higher level of mental comorbidity than the other groups of women, overall study periods, females suffering from AN or Eating disorder not otherwise specified-purging (EDNOS-P) had the highest percentage of psychotropic drug use. More than one in five of these women exhibited symptoms of depression and anxiety during pregnancy, and females suffering from AN also had the greatest rate of frequent psychotropic drug use (i.e., before, during, then after pregnancy) (5.6%) [34].

Women with EDNOS-P or AN were 6.8 and 5.1 instances much more likely to use sedatives or anxiolytics in their postpartum care, respectively, even after controlling for postpartum weight reduction and melancholy and tension signs. The large physical adjustments that accompany childbirth may also provide a specific hassle for women with AN, who are characterized by way of a great fear of gaining weight and a warped perception of frame form. According to certain research studies, having BN during pregnancy increases the probability that it would remit at 18 and 36 months after delivery [14]. The prevalence of preconception psychotropic drug use among ladies with AN (53%) or BED (18%) or all-consuming disorders had been suggested to decrease than those determined in three preceding investigations (96.7%) [35].

The vast use of analgesics earlier than, throughout, and after pregnancy becomes a function of women with BED. But the multivariate analysis revealed that BED become no longer drastically associated with analgesic use throughout pregnancy or postpartum. This locating suggests that different elements, inclusive of depressive and tension symptoms, pain conditions, body mass index, and weight trade in the course of pregnancy and postpartum, may also act as the key motives for analgesic use instead of overeating [36].

All of the eating disorders in our analysis had high rates of gastrointestinal drug usage. This observation may indicate that pregnant females suffering from eating disorders experience more gastrointestinal discomfort than pregnant women without eating disorders, but it also raises several questions. In particular, women with BED used gastrointestinal drugs more frequently during pregnancy (antacids, laxatives, and drugs for gastroesophageal reflux disease (GERD), but not before pregnancy. This finding raises the possibility that bingeing episodes during these times may be more severe or frequent or that pregnancy-related gastrointestinal problems may be more severe as a result of the binge [31,37].

Management of eating disorders in pregnancy

Obstetric clinicians must be aware of warning indicators and assessment strategies to spot them because females suffering from eating disorders may be hesitant to reveal symptoms to medical professionals. Recent research revealed that the newly created diagnostic instrument, the eating disorder examination, can help determine whether a person has an eating disorder. Females suffering from eating disorders can be distinguished from healthy controls using questionnaires from the eating disorder examination about body image, food avoidance, dietary regulations, and dieting habits. We present a method for the treatment of people who are known to have eating disorders or who are suspected of having them. For the treatment of expectant mothers with eating disorders, we advise a team approach that places a focus on constant communication and precise goal setting [38].

## Conclusions

In line with the authors of the reviewed guides, pregnant women tormented by eating disorders are a numerous organization of sufferers. Females stricken by eating disorders onset before pregnancy and those with eating disorders onset at some stage in being pregnant were separated. It turned into highlighted that pregnant ladies are sensitive to external alerts and liable to growing eating issues, which take place after delivery and feature symptoms that continue for years. However, neither the term "pregorexia," which is not yet included in any classification nor the criteria that this illness might fit, were mentioned in the investigations. Additionally, in some individuals with eating disorders before becoming pregnant, symptomatic improvement was seen during the pregnancy, whereas in other cases, on the other hand, perinatal symptom progression was noted. According to recent scientific research, breastfeeding is a reliable method for preserving and enhancing baby health. In any event, it can be said that mothers with any eating disorder subtype are likely to experience more breastfeeding-related issues, which is difficult for both the mother and the child. The child may also experience these negative emotions and communicate their discomfort by acting differently.

Finally, several variables, including a greater maternal age, hypertension and eclampsia, multiparity, hypoxia problems, prematurity or preterm birth (<32 weeks), and being small for gestational age, were observed to have an effect on the beginning of AN in the offspring. Specialists in gynecology, psychiatry, neonatology, and pediatrics must work together throughout disciplines to deal with pregnant women with active eating disorder signs and symptoms or a history of eating disorder, as well as the infants they provide delivery to. Consequently, a better clinical understanding of eating disorder symptomatology during pregnancy is required, and antenatal treatment should include the use of suitable screening methods. Currently, there is a lack of information on eating disorders among pregnant women, particularly in teens, which suggests that further study is needed in this area. We advise close observation of expectant mothers who have an eating disorder, either present or former. Children born to these mothers need to receive attention.
